# Polymer Therapeutics: Biomarkers and New Approaches for Personalized Cancer Treatment

**DOI:** 10.3390/jpm8010006

**Published:** 2018-01-23

**Authors:** Stuart P. Atkinson, Zoraida Andreu, María J. Vicent

**Affiliations:** Polymer Therapeutics Laboratory, Centro de Investigación Príncipe Felipe, Av. Eduardo Primo Yúfera 3, 46012 Valencia, Spain; satkinson@cipf.es (S.P.A.); zandreu@cipf.es (Z.A.)

**Keywords:** biomarkers, polymer therapeutics, tumor-derived exosomes, polymer-based combination therapy, cancer, nanomedicine

## Abstract

Polymer therapeutics (PTs) provides a potentially exciting approach for the treatment of many diseases by enhancing aqueous solubility and altering drug pharmacokinetics at both the whole organism and subcellular level leading to improved therapeutic outcomes. However, the failure of many polymer-drug conjugates in clinical trials suggests that we may need to stratify patients in order to match each patient to the right PT. In this concise review, we hope to assess potential PT-specific biomarkers for cancer treatment, with a focus on new studies, detection methods, new models and the opportunities this knowledge will bring for the development of novel PT-based anti-cancer strategies. We discuss the various “hurdles” that a given PT faces on its passage from the syringe to the tumor (and beyond), including the passage through the bloodstream, tumor targeting, tumor uptake and the intracellular release of the active agent. However, we also discuss other relevant concepts and new considerations in the field, which we hope will provide new insight into the possible applications of PT-related biomarkers.

## 1. Introduction: Polymer Therapeutics and the Requirement for Biomarkers

Polymer Therapeutics (PT) are amongst the most successful polymeric nanomedicines [1,2] with two compounds reaching the US Top 10 selling drugs list; the white blood cell booster Neulasta^®^ (Amgen, Thousand Oaks, CA, USA), a polyethylene glycol (PEG) modified form of the recombinant human granulocyte colony-stimulating factor (G-CSF) analog filgrastim, and the immunomodulatory drug Copaxone^®^ (Teva Pharmaceutical Industries, Petah Tikva, Israel), otherwise known as glatiramer acetate [3]. PT describe complex multicomponent polymeric drugs, polymer-based conjugates and delivery systems, which are considered new chemical entities (NCEs) rather than conventional drug-delivery systems or formulations that entrap, solubilize, or control drug release without resorting to chemical conjugation. Conjugation enhances aqueous solubility and alters drug pharmacokinetics at both the whole organism and subcellular level leading to improved therapeutic outcomes [4,5] and can provide solutions for individualized therapies [6]. Furthermore, PT-based combination therapies allow the simultaneous co-transport of multiple active agents directed to different targets to further enhance therapeutic outcomes [7].

Even given these important improvements over “free drug” formulations and the possibility of enhanced synergistic combination strategies, many PT-based strategies fail at the preclinical or early-clinical trial stage [8]. Indeed, recent figures have shown that around 90% of all compounds entering Phase I clinical trials do not meet with approval and do not enter the market [9]. In the realm of PTs, a self-assembled nanoparticle comprising camptothecin (CPT) covalently conjugated to a linear, cyclodextrin-PEG (CD-PEG) co-polymer (CRLX101) suffered from a poor clinical trial outcome [10], while the anti-cancer paclitaxel (PTX)-polyglutamic acid (PGA) conjugate Opaxio^TM^ (paclitaxel polyglumex; Cell Therapeutics Inc., Seattle, WA, USA) [11] and PEG-irinotecan (etirinotecan pegol) [12] did not achieve the primary clinical endpoint needed to satisfy regulatory approval, underscoring the need to rethink development strategies. 

**Figure 1:**
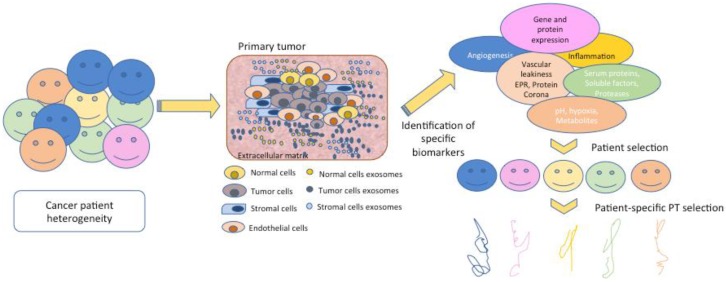
The basis of personalized medicine is the knowledge of the environment, in this case the tumor’s microenvironment in each patient. The basic players of this disease include normal cells, tumor cells, the vasculature and the stroma, but the signals sent by these components and their features are different and give rise to patient-specific tumor heterogeneity. The identification of signals such as membrane receptors, proteases, angiogenic factors, and opsonins among others are key for the design and development of polymer therapeutics (PT) and the selection of those patients with maximal response.

However, evidence exists of the therapeutic success of PTs in specific subsets of patients, suggesting that the stratification of patients into treatment categories according to specific biomarkers may improve therapeutic outcomes. The choice of patients who will benefit and, importantly, the separation of those who will not, will improve treatment-associated success rates, reduce potential side effects in non-responding patients and reduce costs associated with “wasted” PT production and treatment. While this is becoming a generally accepted principle, there is a distinct lack of PT-related biomarkers, although the swathes of data generated from clinical trials may aid in this search (Figure 1) [2].

The most representative example for biomarker utility is for the anti-cancer paclitaxel (PTX)-polyglutamic acid (PGA) conjugate Opaxio^TM^ (paclitaxel polyglumex; Cell Therapeutics Inc.) employed for the treatment of non-small cell lung cancer (NSCLC). Analysis of Phase III trials suggested enhanced activity (increased survival) in females only [11] due to a correlation between higher estrogen levels and increased lysosomal cysteine protease activity (Cathepsin B) required to cleave the PGA backbone and liberate PTX [13,14]. Therefore, PT-based treatment of female NSCLC patients with high estrogen levels may see more success, highlighting both the utility of patient stratification in PT-based treatment and the need to prospectively assess clinical data for additional PT biomarkers. However, a subsequent study in male patients with metastatic prostate cancer discovered poor response to Opaxio^TM^ treatment combined with low-dose transdermal estradiol [15], therefore underscoring the requirement for additional biomarkers.

Within this concise review, we aim to discuss potentially useful biomarkers for PT-based therapies, their detection and their application in cancer treatment. We do note that while non-cancer application of PTs flourish [16], including but not limited to, spinal cord injury [17], neurological diseases [18,19] and enzyme-replacement therapies [20] and many biomarkers will also directly relate to other PT uses, the examples given within this Concise Review will mainly deal with cancer applications. Indeed, the only polymer-drug conjugate currently on the market (Movantik^®^; PEGylated naloxol [AstraZeneca Pharmaceuticals LP, MA, USA]) has been designed to avoid drug transfer through the gut and to remain in the gastrointestinal tract to treat opioid-induced constipation [21]. Additionally, while we hope to discuss biomarkers in relation to PTs, some examples will discuss non-PT nanomedicinal formulations (such as liposomes, inorganic nanoparticles, protein-drug conjugates) where directly relevant to support discussion. While related, we will not discuss antibody-drug conjugates (ADCs) and we refer the reader to an excellent recent review on this exciting class of therapeutics [22]. Finally, we hope to concentrate, where possible, on the most recent advances in this rapidly developing area of study.

## 2. Biomarkers: Current Understanding, Problems and PT-Based Considerations

While many biomarkers represent considerations for most medicines (e.g., presence of pharmacological target, toxicity markers, acquired resistance markers, etc.), many biomarkers will be PT-specific given their pharmacokinetic profile, specially-designed stimuli-sensitive triggering mechanisms, potential for passive targeting and other related considerations.

Current information on biomarkers from preclinical/clinical studies in animal models [23] and analysis of clinical nanomedicines currently in use for cancer treatment in humans [24] suggest that future PT design strategies must take account of constraints imposed by environment factors or “hurdles” that a PT/nanomedicine encounters during its bodily journey from administration to target site to clearance. While navigating these hurdles, we hope to discuss potential biomarkers, their detection and the application of this knowledge, not only to stratify patients but also to improve anti-cancer treatments and guide the future design of more effective PT approaches.

## 3. Hurdle 1: The Bloodstream

The bloodstream generally represents the first hurdle faced by a PT, which are designed to ensure chemical stability, integrity and non-toxicity during blood circulation at physiological conditions until the PT reaches the desired site of action (i.e., a tumor site). However, the final physico-chemical properties of each PT [25] significantly impacts their biological interactions and elicited responses, with important parameters including size, charge, conformation, geometry, composition, polymer-active agent linkers and ligand patterning (See as Nakamura et al. [26] as an example of the importance of linking chemistry and drug nature). We consider some of the most important blood factors considered in PT-based therapeutics, complement activation, anti-polymer antibodies, protein corona formation, blood clotting and opsonization and clearance, as potentially interesting biomarkers. Of note, in many tumor system (or in cancer patients), the thrombus forming coagulation system is highly activated (e.g., pancreatic cancer) and consequently, even low molecular weight (MW) drugs have less access to cancer tissues, so platelet concentration and thrombus presence should must also be considered. Furthermore, PTs must be designed to be stable and non-toxic during the transit through the bloodstream, only becoming active when they reach their site of action.

### 3.1. Complement Activation 

The presence of nanomedicines in the bloodstream (following intravenous (i.v.) injection) can initiate unwanted innate immune responses and in particular complement activation, leading to rapid clearance from the bloodstream and strong inflammatory responses. While early studies of in vitro and in vivo models [27] suggested that *N*-(2-hydroxypropyl)methacrylamide (HPMA) copolymer-anthracycline conjugates did not activate complement [28], preclinical studies have documented complement activation by other PT-related polymers including polylysines, poly(amidoamine) (PAMAM) dendrimers and poly(ethylene imine) [29], PEGs [30] and poloxamers [31]. Additionally, infusion reactions have been clinically observed during i.v. administration of certain polymer-drug conjugates [32,33], leading to Szebeni to warn of a complication described as complement activation-related pseudoallergy (or CARPA) reactions to nanomedicines [34].

A very recent study from Chen et al. studied how complement proteins assembled (or opsonized) on superparamagnetic iron oxide (SPIO) “nanoworms,” consisting of a magnetite-maghemite (Fe_3_O_4_ and γ-Fe_2_O_3_) core and biopolymer dextran shell [35]. The opsonization of nanomedicine by factors such as Complement components enhances nanomedicine recognition and clearance from the bloodstream by the mononuclear phagocyte system (MPS) (also known as the reticuloendothelial system, RES) and may contribute to the infusion-related adverse effects discussed above. Interestingly, this new study found that an alternative pathway (AP) complement component (C3) bound to the blood proteins absorbed to the nanoparticle (known as the protein corona) rather than the nanoparticle itself and underwent dynamic exchange in vitro and in vivo. This suggests that controlling protein corona composition may be key for the control of complement activation (discussed in detail below). 

The application of relevant animal models to test complement-mediated hypersensitivity of specific PTs has proven highly useful [27,36,37]; however, assessments for each patient may be required to fully negate the potential for PT-mediated complement activation. Can we analyze human blood samples to assess the potential for complement activation in response to different PTs? Furthermore, for patients stratified into a high-risk category; can we redesign or reformulate PTs to produce a more muted complement response [38]? A recent study by Benasutti et al. studied the variability of complement C3 binding to preclinical and clinical nanoparticles in the general population, including highly PEGylated liposomal doxorubicin (LipoDox, Sun Pharmaceutical Industries Ltd. Mumbai, India) and minimally PEGylated liposomal irinotecan (Onivyde, Merrimack, MA, USA) [39]. Overall, the study highlighted the variability of C3 opsonization in the general population and the fact that an individual’s response toward one nanoparticle cannot be reliably predicted based on another nanoparticle. 

Studies such as these suggest that patient-specific and nanomedicine-specific assessments of complement binding and activation may be required for safe and effective PT treatment. By assessing blood samples, we may be able to stratify patients into those more likely to have a muted complement response following administration of a given PT and thereby enhance therapeutic outcome.

### 3.2. Anti-Polymer Antibodies

The potential for antibody production in response to treatment based on polymeric materials is typified by the ongoing study of how PEG-conjugate immunogenicity or patient immune reactions in response to pre-existing anti-PEG antibodies can influence clinical performance [40,41,42]. Pre-existing antibodies and/or conjugate immunogenicity can impair efficacy due to rapid blood clearance or impact safety by producing allergic reactions. Population wide studies support the presence of anti-PEG antibodies (IgM and IgG) in a percentage of the population [43,44] with an interesting correlation observed between antibody titer and patient age but not with gender or race, attributed to increasing human exposure to PEG in cosmetics, medicines and various consumer products. Therefore, effective and safe treatment strategies using PEG-related PTs must first screen patients for the presence of anti-PEG antibodies as a biomarker for reduced PT therapeutic activity. Furthermore, such approaches highlight the age of the patient as a potentially vital parameter.

Additional studies have shown the potential for antibody-generation in response to other polymers, including PMOX (poly(2-methyl-2-oxazoline)) [45], so underlining the importance of early monitoring of immunogenicity (IgG and IgM) as a biomarker for any new PT being considered for parental treatment. Furthermore, these findings also suggest that limited immunosuppression may also enhance the function of certain PTs in patients with high blood titers of PT-directed antibodies.

This again could indicate that blood based examinations may allow segregation of those patients with low/high antibody titers and guide choice of polymer before the choice of PT is made.

### 3.3. Protein Corona Formation/Opsonization

On its journey through the bloodstream, individual PTs will interact with a wide range of factors, leading to the formation of an adsorbed protein layer known as the protein corona [46,47] and opsonization, which can lead to enhanced PT clearance from the bloodstream, altered targeting (both inhibition and promotion) and the induction of toxicity [47]. Coronal factors include albumin, immunoglobulin G (IgG), transferrin, fibrinogen and apolipoproteins, while antibodies, complement proteins and circulating proteins such as pentraxins, collectins and ficolins can all act as opsonizing agents. For a wider aspect on the importance of the protein corona to nanomedicine, we direct the reader to a recent excellent review [48].

The formation of the protein corona not only depends on the type of polymer used [49,50] but also the specific physiological environments encountered [51], the duration of exposure [52,53] and also on the state of the patient being treated [54]. The latter study demonstrated that while protein corona formation on various nanomedicines displayed similarities between control and breast cancer patients, blood cancer patients and smokers demonstrated differing protein corona patterns in comparison to healthy controls [54]. Furthermore, genetic background, sex, life-style and race also strongly influence plasma protein levels in healthy individuals [55,56], which all contribute to patient-specific corona formation. 

As mentioned, the formation of the protein corona on PT formulations can significantly affect PT targeting and, therefore overall efficiency. Meister et al. discovered that polylactide (PLA) nanoparticles containing the Aβ42 lowering drug flurbiprofen (as a potential therapy for Alzheimer’s disease (AD)) became quickly decorated with bioactive proteins, including apolipoprotein E, which actually aided the passage of the nanoparticle through the blood-brain-barrier [57]. Furthermore, a study by Papi et al. discovered that protein corona formation also increased cellular uptake of PEGylated liposomal irinotecan in the pancreas ductal adenocarcinoma cell line (PANC-1) [58]. Therefore, we may be able to take advantage of protein corona formation to increase the drug loading capacity, to manipulate pharmokinetics and distribution and inhibit toxicity [59]. However, studies in related nanomedicines have suggested that dynamic protein corona formation modulates the pharmacological and toxicological profile of nanomedicine, leading to unpredictable alterations to functionality [60]. Furthermore, the protein corona can also interrupt receptor-mediated “active” targeting of nanomedicines [61,62] (discussed in more detail below).

Therefore, can we now seek to test patients’ blood (or other relevant biological media) for patient-specific, disease-specific and PT-specific corona and opsonization patterns (as well as other parameters discussed above) in response to various PTs in order to understand which patients are likely to benefit? 

We now have models to understand how the protein corona affects nanomedicine interactions with cells [63,64], although this has been limited to in vitro assessments, which, for the protein corona, do not correlate well with in vivo results [65]. However, recent research has taken a step forward in this regard, with a report of different widely utilized polymers PEG, poly(HPMA) (pHPMA) and poly(methacrylic acid) (PMA) templated onto mesoporous silica nanoparticles for wide-range ex vivo testing in whole cells, rat models and whole human blood [66]. One can envisage patient testing using a wider range of such “test” nanomedicines (with or without functional groups/linkers/targeting moieties etc.) to assess interactions with blood samples to choose the (potentially) “best” polymer for a PT based on the parameters discussed above. Additionally, one report studying the biodistribution of PEG-based particles highlights ex vivo assays on whole human blood as a more sensitive and relevant than traditional in vitro cell-line based assays for predicting in vivo circulation behavior [67]. However, a wider variety of molecular species has been observed for in vivo formed protein corona [68], which may be due to the absence of factors such as blood flow, circulating and endothelial lining cells and immune responses from ex vivo experiments. These data point to an in vivo testing regimen as the most likely to provide faithful biomarker parameters to be employed in PT-based approaches.

Perhaps a simpler method of predicating the potential for protein corona formation and opsonization may be a simple “first-pass” assessment of proteins in patient blood samples. Given the routine assessment of many of these factors in patients undergoing treatment, we may already have sufficient clinical data to correlate levels or patterns of blood protein expression and PT success or failure and/use as a biomarker. Furthermore, this may represent a cheap, efficient and rapid means to safely stratify patients into treatment groups.

But what about patients stratified into “non-responders” based on these biomarkers? There currently exist strategies that may help patients who display high levels of inhibitory coronal/opsonin factors and blood clearance/mistargeting of a given PT. Strategies include “stealth” coatings of PEG that delays immune clearance [69] and labeling nanomedicines with minimal “self” peptides [70] or even components of cell membranes [71,72,73]. Interestingly, a new in vivo study has shown that PEG density on the surface of poly(ethylene glycol)-*b*-poly(lactic-co-glycolic acid) (PEG–PLGA) nanoparticles is a key determinant of their early clearance in vivo in a mouse model, determining an optimal PEG density threshold irrespective of size [74].

A very recent study has presented an even more attractive solution to the problem of nanomedicine clearance by resident macrophages, via the application of the clinically approved antimalarial agent chloroquine rather than through modifications of the nanomedicine under consideration [75]. This straightforward approach inhibited nanomedicine endocytosis by macrophages, reduced nanomedicine accumulation in the liver and spleen, enhanced nanomedicine blood circulation times and improved the delivery of non-PEGylated liposomes to tumors and the site-specific localization of silicon particles in the lungs. Could chloroquine “pre-conditioning” represent a simple and easy means to enhance PT delivery in patients that present with an “overactive” MPS and do away with the need for the conjugation of moieties such as PEG, which carries certain potential side effects (discussed later)? The authors do note that chloroquine treatment may weaken the immune system of the patient and also affect tumor-associated macrophages (TAMs) that aid in the intratumoral uptake of some nanomedicinal formulations (discussed in more detail below) suggesting that a fine equilibrium may need to be found to optimize treatment.

### 3.4. Blood Clotting

Blood clotting may also represent an additional concern regarding the introduction of PTs into the bloodstream. Negatively charged polymers may activate coagulation factor XII (or Hageman factor), which represents the starting point for the clotting cascade and may initiate an unwanted response. Therefore, the implementation of clotting assessments from patient blood samples may represent an important step within the safety analysis profiling of a PT and may direct the implementation of a less-negatively charged polymer in some patients. 

## 4. Hurdle 2: Targeting the Tumor and Tumor Uptake

The next hurdle faced by a given PT is the voyage through the bloodstream to the tumor and then passage from the bloodstream into the tumor site (we note that we concentrate here on the application of PTs to solid tumors, even given reports of efficient PT-based therapies for leukemia [76,77]). Tumor targeting strategies for PTs include the tumor specific uptake of PTs of a certain size by the enhanced permeability and retention (EPR) effect (“passive” targeting) [78,79] or the addition of specific ligands or molecules to the PT to target them to specific tumor-targets (“active” targeting) [80]. Once a given PT has endured this epic journey, the following hurdle is to efficiently move into the cell, a process regulated by numerous factors. Which of these many parameters will represent potentially useful biomarkers?

### 4.1. Passive Targeting by the EPR Effect

PTs of specific sizes passively accumulate in tumors due to the EPR effect, which arises as a consequence of leaky tumor vasculature and poor lymphatic drainage. Many have sought to take advantage of the EPR effect instead of employing molecular targeting, by assuming that EPR represents a universal factor in solid cancers that would allow us to overcome tumor heterogeneity (e.g., related to mutation load). However, not all tumor types display EPR-mediated targeting and the process can be tumor-size dependent. The tumor vasculature is a dynamic, inflammatory environment with both vascular permeability and blood velocity complex and kinetically variable from segment to segment [81]. Recent studies employing intravital confocal laser scanning microscopy have described the transient and stochastic occurrence of ‘dynamic vents’ associated with leaky blood vessels, underscoring the heterogeneity and evolution of the vasculature and hence, altered targeting, by the EPR effect [82]. Furthermore, metastatic sites and the primary tumor will also differ with regard to their potential for passive targeting [83]. 

The first evidence for heterogeneity of EPR-mediated targeting came over 20 years ago following administration of a ^131^I-labelled HPMA-copolymer doxorubicin (Dox) [84] and several clinical studies (using liposomal formulations of chemotherapeutic agents) have suggested the value of stratifying subpopulations of cancer patients according to their likelihood of EPR-mediated targeting [85,86,87]. Other techniques used to assess EPR include tumor imaging using iodine-labeling [32,84,88], including the well-known contract agent Lipiodol [78], technetium-labeling [87] and more recently, copper-labeling [89], highlighting both the relative ease of assessing and the general importance of this biomarker. This measurement would encompass alterations to passive targeting mediated by heterogeneity in tumor size, stage, metastasis and vascularity, although other potential biomarkers influencing EPR include gene/protein/cell biomarkers, such as levels of vascular/angiogenic markers, inflammatory mediators/inflammation, as well as disease specific parameters. Additionally, the overall state of the circulatory system is of vital importance to the EPR effect [78,83] as the presence of ischemia, the formation of thrombi, or vascular embolism that can suppress blood flow can nullify the EPR effect and severely limit passive targeting of PTs. Of note, tumors passively accumulate nanomedicines of different sizes [90] suggesting that testing EPR with different sized PTs/probes may provide information that will optimize tumor accumulation/tumor uptake of a therapeutic PT.

A recent study from Rogers et al. demonstrated the utility of 100 nm iridium-labelled gold nanoparticles to track the flow of blood through micrometer-sized blood vessels in normal mouse tissues using luminescence imaging [91]. The potential for in vivo high-resolution visualization and monitoring of blood flow based on small fluorescently labelled nanoparticles in cancer patients could provide valuable biomarker data on the potential for passive targeting and pharmacokinetic parameters. 

Therefore, treating patients with well-vascularized tumors with larger PTs may be an optimal treatment strategy; but how do we approach those tumors/patients with poor EPR-based parameters? 

Interestingly, there exist tested approaches to enhance passive targeting in patients stratified as non-responsive to EPR or to overcome the issue of EPR heterogeneity to a great extent. Strategies include treatment with nitric oxide (NO) [92,93], angiotensin II [94,95] and carbon monoxide [96,97], amongst many others (Reviewed in Maeda et al. [78]), which lead to enhanced drug delivery by potentiating the EPR effect. Interestingly, Šírová et al. employed NO donors linked to an HPMA carrier through a stable bond or through a hydrolytically degradable, pH sensitive bond to specifically enhance HPMA copolymer-Dox treatment of mouse tumors influenced by the EPR effect [92]. Of note, NO co-treatment did not potentiate in vitro toxicity of HPMA copolymer-Dox and had no additional effects in tumors not affected by the EPR effect, indicating the dominant effect of EPR-mediated uptake. Furthermore, benefit of NO-releasing agents has also been shown in a clinical setting using conventional low MW anticancer agents [98,99]. The levels of some of these factors in patients may therefore also relate to the likelihood of success of PT-based therapies.

A more recent study also demonstrated that a single, low dose of radiation therapy induced increased permeability and enhanced delivery of liposomal irinotecan to tumors in a tumor-associated macrophage (TAM)-associated mechanism [100]. Importantly, this study suggests that strategies seeking to improve nanotherapies should concentrate on modifying the local tumor microenvironment (TME) and not the nanomedicine itself and so may represent a more relevant and easier to accomplish means of enhancing PT-mediated anti-cancer therapies. The existence and number of TAMs as a potentially interesting biomarker will be discussed further below.

Levels of vascular endothelial growth factor receptor-2 (VEGFR2) may also represent an interesting biomarker with regards to the size of the PT employed in cancer treatments. Studies have found that blocking VEGFR2 (and promoting repair of abnormal vessels) can improve tumor-delivery of small nanomedicines (diameter, 12 nm) and hinder delivery of larger nanomedicines (diameter, 125 nm) [101]. Not only this represent an interesting biomarker but also a means to enhance delivery of PTs of smaller size. Additionally, blood markers for tumor-related angiogenesis could also provide interesting biomarker information, as levels of distinct factors may provide information regarding the blood flow and the capacity for EPR-mediated targeting [102,103]. 

### 4.2. Active Targeting

Active targeting strategies can be employed instead of, or to augment, passive targeting of PTs and approaches include surface modification of PTs with ligands directed against disease/cell-specific receptors [80]. A recent example of active targeting took advantage of the tumor-specific overexpression of riboflavin transporters (RFTs) and the riboflavin carrier protein (RCP) to enhance the delivery of branched PEG polymers decorated with riboflavin moieties [104]. Of note, polymer size determined uptake by different tumor cell compartments, with smaller conjugates efficiently targeting cancer cells and larger conjugates efficiently targeting TAMs. Furthermore, active targeting may also be used in an attempt to target a given PT to multiple cell types, such as tumor cells and angiogenic endothelial cells via integrin binding [105]. Interestingly, while not initially designed with active targeting in mind, clinical studies of Abraxane treatment in head and neck cancer patients suggests that response to therapy correlates to levels of the extracellular matrix glycoprotein albumin-binding protein SPARC [106,107]. Therefore, a primary biomarker for active targeting should be assessment of the presence of said receptors. 

However, receptor expression is often variable and varies with stage of disease and TME, suggesting that dynamic imaging techniques may identify patients at the stage of disease most likely to respond to targeted therapies [108]. Different polymer/polymer formulations may also interact with specific cell surface receptors and correlate with clinical response, although such correlations have not yet been reported.

### 4.3. Tumor Microenvironment (TME)

Components of the TME play a key role in controlling the access of PTs to tumors [109] and many barrier components are currently known to exist. Factors include abnormal and heterogeneous extracellular matrix (ECM), blood flow and vessel permeability, which affect the delivery, penetration and homogeneous distribution of nanomedicines in tumors [110], thereby affecting both passive and active targeting.

The ECM component, hyaluronic acid (HA), represents a major obstacle for the tumor access of PTs and so underscores the need to assess TME-ECM components as a biomarker for PT treatment. Recognition of this obstacle/biomarker has led to the development of enhanced nanomedicine strategies. The degradation of HA via hyaluronidase treatment increases tumor uptake of liposomal Dox [111] and the free form of gemcitabine (GEM) [112], while the application of PEG-hyaluronidase conjugate (PEGPH20) in a Phase II trial of Abraxane in untreated stage IV metastatic pancreatic ductal adenocarcinoma correlates treatment success with HA tumor levels [113]. This suggests that ECM analysis may allow treatment strategies to be altered to improve PT-mediated anti-cancer therapy.

Another important component of the TME are TAMs, which have been described as reservoirs that gradually release their “payload” to neighboring tumor cells [114]. Therefore, the presence or absence of TAMs, which can be measured using magnetic resonance imaging (MRI) of clinically applicable ferumoxytol SPIO nanoparticles [115], may correlate to PT success. A recent study employing high-resolution intravital imaging microscopy demonstrated that ferumoxytol colocalized to cancer cells and TAMs and could be used to predict the anti-cancer efficacy of a 90 nm docetaxel-encapsulated PEG-PLGA nanomedicinal formulation [116]. Therefore, routine ferumoxytol assessments in cancer patients may provide predictive data regarding passive targeting and tumor cell/TAM uptake (see below) and therefore, allow the stratification of patients for PT treatment. Interestingly, a study has described TAM-specific targeting in primary and metastatic breast cancer using HPMA-copolymer nanocarriers as a possible therapeutic approach [117].

A further interesting concept with regards to cancer treatment and biomarkers may be the presence of therapeutically resistant, potentially metastatic, tumor-initiating cancer stem cells (CSCs). As with most conventional anti-cancer treatments, we must consider if we are targeting the symptoms (bulk tumor cells) or the route cause (CSCs) and so, the likelihood of encountering CSCs in a given tumor may provide extra insight towards the choice of PT for patient treatment as well as the design of targeted therapies. The presence of CSCs can be assessed (and targeted) using cell surface markers, such as CD44 and these may be useful in choosing/formulating specifically targeted PTs for personalized treatments [118,119]. Of note, Kopecek et al. demonstrated the preferential prostate CSC toxicity of an HPMA copolymer-cyclopamine conjugate (P-CYP) [120].

Overall, while the TME represent a highly dynamic multi-factorial environment, studies have reported some potentially important biomarkers (HA levels, TAM levels) that may prove useful in the stratification of patients.

### 4.4. Tumor Uptake

Following passive and/or active targeting to the cell, the next hurdle facing PTs is uptake and penetration into tumor cells/tumors themselves [121]. A primary biomarker may be evidence of transport mechanisms that permit PT uptake and accumulation within tumor cells. Specific examples include the previously mentioned case of therapeutic strategies employing riboflavin-targeted branched PEG polymers (which can be conjugated with active agents); if we cannot detect the expression of RFTs/RCPs in the tumor target, is there a case to go ahead with this specific treatment approach [104]?

With regards to uptake in more general terms, studies have highlighted tumor-specific abnormalities in endocytic internalization (e.g., Rab proteins) [122] and trafficking pathways [123], which we may be able to detect via tumor biopsies and use as a biomarker for PT-therapy success. However, these biomarkers may not only act as indicators of response but also to the evolution of acquired resistance via the expression of cancer multidrug resistance (MDR)-associated markers [124], which may in turn represent a testable biomarker [125,126].

With this knowledge, some have begun to design polymeric nanomedicines with enhanced tumor uptake and penetration in mind; Guo et al. reported on the coating of polymeric micelles with poly(glutamic acid)-*g*-methoxyl-poly(ethylene glycol) (pGlu-*g*-mPEG) in a delayed charge reversal strategy that allows tumor uptake and enhances tumor penetration [127]. Can this strategy help to promote PT uptake and penetration in patients with low tumor uptake biomarkers scores?

The size and stage of tumor can affect PT uptake, as shown in a study of PEGylated liposomes in which smaller tumors displayed higher uptake [128] and thus must be taken into consideration when choosing specific PT-treatments. Are small PTs best to treat newly developed tumors and micrometastasis, while other larger PTs may be better suited for the treatment of larger more developed tumors? We may be able to stratify patients with enhanced uptake in tumors via the application of tumor-specific fluorescent probes [129].

Interestingly, a study has described how a mutation to the *KRAS* gene in pancreatic cancer cells display elevated levels of macropinocytosis, a form of endocytosis, of proteins such as albumin [130], a fact which may be behind the recent success of Abraxane in treating advanced pancreatic cancer [131]. This raises the question as to if oncogenic mutations correlate to increased endocytosis of PTs and can be used as biomarkers for PT therapeutic response. Further analysis of clinical trial data may highlight further links between gene mutations and the therapeutic potential of a given PT, as mutations will importantly affect vital aspects other than endocytotic uptake.

## 5. Hurdle 3: Intracellular Release of Active Agent

Studies recognizing differences between normal and disease cells and tissues, including pH, redox state and levels of specific enzymes, have led to the clever design of PTs that release their therapeutic payload in response to tumor-specific triggers. Therefore, the precise measurement of these parameters in a patient-specific manner may allow us to identify patients that may respond better to pH-triggered PTs than to enzyme-triggered PTs. However, a primary biomarker must also be the presence of the pharmacological target of the payload itself in relevant cases.

### 5.1. Enzymatic Triggers

Following endocytic uptake and travel to the lysosome, specific enzymes can cleave the polymeric backbone and/or specially designed linking moieties between the polymer and the active agent to release and activate said active agent. Well studied PTs, including Opaxio^TM^ [132], PGA conjugates and HPMA copolymer conjugates, have taken advantage of the activity of the cathepsin protease family [133] and especially the tumor-associated overexpression of cathepsin B [134]. Therefore, the presence of a high level of this enzyme represent an effective biomarker for response to related PTs. 

Other important enzymes for polymer/linker degradation include amylase, which degrades PTs containing dextrin (e.g., [135]) and hydroxyethyl starch (HES) (e.g., [136]), lipase, which degrades poly-caprolactone based micelles [137] and matrix metalloproteinases (MMPs), which can liberate drugs from specially designed micelles [138]. To take advantage of overexpressed enzymes, many PTs have been designed with protease-sensitive linker oligopeptide moieties (e.g., GFLG (Gly-Phe-Leu-Gly) and GLFG (Gly-Leu-Phe-Gly)) designed to be stable in the blood but rapidly cleaved by lysosomal enzymes (cathepsin B or D and others) within the tumor stroma [139]. A recent study by Zhang et al. developed 80–100 nm nanoparticles based on PEGylated poly(L-lysine) dendrimers conjugated with GFLG-GEM for specific drug release under cathepsin B for the treatment of breast cancer [140].

The relative importance of such enzymes as a biomarker was demonstrated in preclinical studies of HPMA-copolymer-Dox treatment of human and mouse tumors; one study highlighted greater variation in drug release than the variation in EPR-mediated targeting [141], while another confirmed a pivotal role for cathepsin B-mediated drug release in the antitumor activity observed [142].

An exciting new study from Shabat et al. may now allow detection and assessment of cathepsin B levels from tumor biopsy samples as a PT biomarker [143]. The authors describe the first demonstration of chemiluminescence cell images obtained by a probe for a natively expressed endogenous enzyme and highlight the possible application of their strategy for other relevant proteases, as noted above.

Specifically increased enzyme levels in tumor cells, such as those observed in human colon cancer compared to normal tissues [144], have also led to the development of polymer-based companion diagnostic strategies that have reached clinical trials [145,146]. Mito et al. reported on the application of a cathepsin-activated fluorescent probe with a polymeric backbone (VM249), which can be applied to detect microscopic residual soft tissue sarcoma (STS) in the tumor bed of mice during gross total resection [147]. Cuneo et al. found that the same probe could differentiate normal and tumor tissues following radiation therapy in mice and canines [148]. Further similar clinical applied probes include cathepsin/MMP-sensitive poly-lysine-based near-infrared fluorescence (NIRF) probes [149,150] for the early and effective detection of cancers of the gastrointestinal tract [151] and pan-cathepsin-sensitive PEG-based probe for the detection of STS and breast cancer [129,152].

Overall, the presence of specific enzymes represents an important and well-recognized biomarker for PT-based therapies, with the clinical application of enzyme-responsive probes to detect tumorigenic cells demonstrating the full potential of this biomarker. However, more detailed studies using some of the advanced techniques discussed may allow us to identity patients more or less likely to respond to enzyme-triggered PTs.

### 5.2. pH/Redox Triggers

Both the TME and certain intracellular organelles present with lower pH and higher reductive potential than normal, making them important considerations for the design of tumor-specific PTs and a potential biomarker. To this end, recent stimuli-responsive nanomaterials being investigated include pH- [153] and redox-sensitive [154,155] nanomedicines. 

PTs have also been designed with pH-responsive linkers, which include the incorporation of hydrazone, acetal, *cis*-acotinyl, Schiff-base and β-thiopropionate moieties between the polymer mainchain and the active agent (e.g., [26,153,156]), while pH-sensitive polyacetals (PAs) have also been used in the construction of polymer-masking-unmasking-protein therapy (PUMPT) strategies [157] and as a drug carrier for prostate cancer treatment [158]. In PUMPT-based strategies, a PA-chain protects the active agent (e.g., protein, small drug) from degradation (“masking”) in the neutral pH of the blood stream but releases the active agent via PA-chain degradation in the presence of the acidic pH of the tumor site/lysosome (“unmasking”) [157]. The latter study [158] sought to employ metabolomic assessments of prostate cancer cells in response to hypoxia and to pharmacological HIF-1α inhibition by free diethylstilbestrol (DES) or PA-conjugated DES. Overall, this study revealed a number of unanticipated metabolic changes in response to PA-conjugated DES, which could represent potentially interesting biomarkers. 

Redox responsive PTs include dual-responsive Dox loaded micelles of PEG-polycarbonate diblock copolymers functionalized with disulfide bonds and pH-responsive carboxylic acid groups [159], PEG-modified redox-responsive chain-shattering polymeric therapeutic (CSPT) drug carriers [160] and additional PUMPT strategies [161].

Overall, the pH and redox state of cells/tumors represent potentially exciting biomarkers for PT treatment, as the differential sensitivity of pH/redox-responsive linkers may allow personalized PT therapies.

Interestingly, new studies have described means to assess pH and redox state at the single cell level. Perry et al. recently applied resonance Raman spectroscopy for continuous monitoring of redox state on the epicardial surface of the heart [162]. In this study, the authors quantify the reduced fraction of specific electron transport chain cytochromes, or the resonance Raman reduced mitochondrial ratio (3RMR) that the authors suggest may permit the real-time analysis of organ-specific oxygen delivery. The development of reversible reaction-based fluorescent probe, such as RealThiol (RT) [163], that can quantitatively monitor real-time glutathione dynamics in living cells may also find use in delineating this biomarker in vivo. Additionally, both Hu et al. [164] and Wang et al. [165] recently described techniques which allow accurate and sensitive pH detection on the single-cell or sub-cell level indicating the ability to assess PT-related biomarkers in great detail. Hu et al. [164] employed an innovative combination of UV-Vis microspectroscopy and common pH indicators, while Wang et al. [165] applied ultra pH-sensitive fluorescent nanoprobes that display tunable, exponential fluorescence activation on encountering subtle, physiologically relevant pH transitions. 

## 6. Hurdle 4: Further Concepts

What other parameters may act as useful biomarkers for in PT-based therapies? Certainly, gender, race, age, disease state, comorbidities and exposure to other treatment can influence the success of PT-based therapies, as they can with most treatments for any treatment approach. A recent clinical study found that age correlated to altered exposure levels of a CPT analogue delivered by PEGylated liposomes and also highlighted the influence of prior exposure to PEGylated liposomal Dox [166]. Another interesting study also reported increased activity of a PEG-irinotecan conjugate in breast cancer treatment when associated with metastasis to the brain [12]. 

For PT treatment, as for all drugs, we must also study how toxic any given treatment may be in non-tumor tissues [167] driven by unwanted targeting/accumulation due to a range of factors. Interestingly, recently developed techniques detected free PEG or PEG-conjugates in tissues and plasma [168,169] and can be used to quantify pharmacokinetics and tissue distribution to identify at risk tissues before moving into first-in-man studies. Furthermore, a study from Griffin et al. employed in vivo intravital microscopy to begin to understand the skin accumulation and toxicity of non-PEGylated and PEGylated liposomes after systemic injection into mice [170]. Studies such as this with PTs may uncover more on pharmacokinetics, aggregation, normal tissue toxicity and again, provide important information regarding future clinical application.

## 7. Hurdle 5: Moving towards the Finishing Line: Improved Detection, Better Models, New Biomarkers and New PT-Based Therapies

The development of new technologies has accompanied the development of new PT strategies and these may allow us to assess potential biomarkers for PT-success in greater detail. Single tumor cell analyses of RNA and protein expression will allow us to deeply understand targets for PT-based therapies, assess tumor heterogeneity, uncover new biomarkers and allow us to personalize treatments [171,172]. The detailed analysis of single-cell metabolomics [173,174] may also highlight new targets, new triggers and mechanisms of therapeutic resistance for PT treatment [158]. Indeed, recent studies in our laboratory have shown distinct metabolic changes when comparing the treatment of breast cancer cells in vitro and in vivo in response to a chemotherapeutic anthracycline drug (Dox) and an HPMA copolymer-conjugated form (HPMA-Dox) [175]. Studies such as this, coupled with new advances in the understanding of cancer metabolism [176], may provide essential biomarkers for PT-based therapeutic approaches.

Multimodal imaging and treatment can inform on PT treated patient-specific outcomes on the fly [177] to provide early evidence of response (an important biomarker), although the study of long-term PT treatment may also provide important data [178]. Indeed, one major problem noted with long-term PEG-based treatments is the risk of intracellular accumulation and vacuolation [179,180]; however, new magnetic resonance spectroscopy (MRS) techniques that measure PEG concentrations in vivo, as well as previously noted approaches [168,169] may represent a feasible imaging-based method to detect this important biomarker [181]. 

The development and application of appropriate model systems will also be of great importance moving forward, given the noted lack of correlations between in vitro analysis and in vivo outcomes. Indeed, many nanomedicines interfere in biomarker assays classically applied for small molecule drugs [182]. As an example, many in vitro analyses of PT toxicity, uptake etc., employ two-dimensional cell culture where three-dimensional tumor spheroid cultures represent a better model system to test new chemotherapeutic agents [183] and model patterns of chemoresistance and tumor recurrence [184]. Furthermore, many preclinical studies rely on animal models, which do not fully recapitulate human biology, tumor development and therapeutic response. 

**Figure 2:**
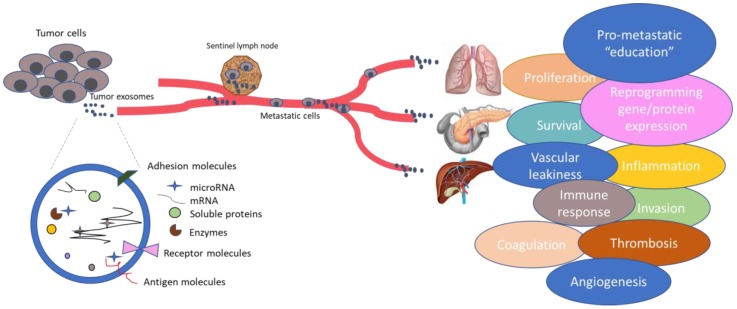
Exosomes released from cancer cells contain important information regarding cancer progression and metastasis. Exosomes are pleiotropic, as they are involved in many steps of cancer, including angiogenesis, invasion, microenvironment education, drug resistance, etc. and represent an important source of biomarkers. Therefore, exosomes could be highly important for the development of future therapies.

Of specific interest to both PT-based therapies, cancer treatment and biomarker research is the ever widening field of exosomes [185,186], now considered a key platform for intercellular communication (Figure 2). Tumor cells release large numbers of exosomes enriched in proteins, mRNAs and microRNAs involved in the several steps of cancer progression and metastasis. Tumor exosome integrins can determine organotropic metastasis [187] and so assessment of cell surface receptors such as integrins can be used to predict future sites of metastasis in cancer patients [188]; could we also take advantage of this information to guide PT-based therapies? Several studies have described their role in chemotherapy cancer resistance, as exosomes can sequester chemotherapeutic drugs in melanoma [189] and ovarian cancer [190]. Can they also sequester polymer-conjugated forms? Analysis of exosomes and exosomal biomarkers is made easy by their and minimally invasive isolation from body fluids and could be used to select and establish patient groups to predict therapy response from personalized PTs [191,192]. Interestingly, some studies have even sought to harness the inherent characteristics of exosomes, such protection of their cargo from degradation by proteases and RNases, for targeted drug delivery strategies in cancer [193,194,195], which overcome limitations present in other drug delivery systems [196]. Could exosomal strategies further enhance PT-based cancer therapeutics?

## 8. Conclusions: Last of the Hurdles but Only the Beginning of the Race

While this review hopes to have covered many potential biomarkers (See Table 1), the identification of the most important of these is left open to the consideration of the reader. Only intense research, collection and assessment of clinical data and the application of new technologies will identify the optimal biomarker or pattern of biomarkers for PT-based therapies. Indeed, the clinical detection of many biomarkers discussed in this concise review is not currently feasible or impossible to apply on a patient-to-patient basis due to current technological limitations or time and labor costs. However, we do have an understanding of certain biomarker parameters that should guide the design of new PT-based approaches. A well-vascularized tumor displaying signs of chemotherapeutic resistance may guide the design of a large PT-based combination therapy carrying a synergistic combination of drugs conjugated via pH/enzyme labile linkers. Likewise, high titers of anti-PEG antibodies in a patient with poor EPR tumor characteristics may guide the design of a smaller HPMA-derived PT modified to boost tumor-specific active targeting. However, with time, many of the discussed advanced technologies will be sufficiently simple and cost-effective to be taken advantage of in a clinical setting, much like the enzyme-responsive probes to guide cancer-related surgical procedures.

## Figures and Tables

**Table 1:** A summary of Hurdles and Related Biomarkers.

Hurdle	Biomarker	References
Bloodstream	Complement Activation	[27,28,29,30,31,32,33,34]
	Anti-polymer Antibodies	[40,41,42,43,44,45]
	Protein Corona/Opsonization	[49,50,51,52,53]
Tumor Targeting	Passive Targeting Parameters	[81,82,83,84,85,86,87]
	Active Targeting Parameters	[80,104,105]
	Tumor Microenvironmental Factors	[113,115,120]
	Tumor Uptake	[122,123,128]
Intracellular Release	Enzyme Levels	[134,141,142]
	pH	[26,153,156,157]
	Redox Status	[154,155,159,160,161]
Additional Hurdles	Gene Mutations	[130]
	Cell Metabolites	[158,175]
	Age	[55,56,166]
	Sex	[55,56]
	Prior PT Treatments	[166]
	Health/Comorbidities	[12,54]
	Healthy Tissue Toxicity	[167]
	Exosomes	[189]
